# Effects of Harvest Maturity on the Chemical and Energetic Properties of Corn Stover Biomass Combustion

**DOI:** 10.3390/ma15082831

**Published:** 2022-04-12

**Authors:** Dawid Wojcieszak, Jacek Przybył, Łukasz Czajkowski, Jerzy Majka, Artur Pawłowski

**Affiliations:** 1 Department of Biosystems Engineering, Poznań University of Life Sciences, ul. Wojska Polskiego 50, 60-627 Poznań, Poland; jacek.przybyl@up.poznan.pl (J.P.); artur.pawlowski@up.poznan.pl (A.P.); 2 Department of Wood Science and Thermal Techniques, Faculty of Forestry and Wood Technology, Poznań University of Life Sciences, Wojska Polskiego 38/42, 60-637 Poznań, Poland; lukasz.czajkowski@up.poznan.pl (Ł.C.); jerzy.majka@up.poznan.pl (J.M.)

**Keywords:** corn stover, corn cobs, high heat value, combustion, corn stover fraction

## Abstract

Over the last decade, there has been increased interest in applying biomass as a raw material for producing biofuels used for thermochemical conversions. Extensive use of biomass could lead to controversial competition for arable land, water, and food; therefore, only waste materials and agricultural by-products and residues should be used to produce biofuels. One suitable by-product of agricultural production is crop residue from the harvest of maize for grain (corn stover). The harvest residues of corn stover consist of four fractions, i.e., husks, leaves, cobs, and stalks, which are structurally and morphologically distinct. The aim of the study was to determine the effect of selected maize cultivars with distinct FAO (Food and Agriculture Organization of the United Nations) earliness classifications on the chemical and energetic properties of their corn cob cores. We determined the chemical properties based on elemental analysis, and the energy properties based on the heat of combustion and calorific values. The content of ash and volatile compounds in the corn cobs were also determined. The results indicated that the heat of combustion of fresh and seasoned corn cob cores ranged from 7.62–10.79 MJ/kg and 16.19–16.53 MJ/kg, respectively. The heat of combustion and calorific value of corn cob cores in the fresh state differed significantly and were strongly correlated with maize cultivars with distinct FAO earliness.

## 1. Introduction

The extensive use of fossil fuels for energy production has raised concerns about their harmful effects on the environment and the related future energy supply [1,2,3,4]. As a result, interest in developing alternative, environmentally-friendly energy sources continues to grow [5]. Over the last decade, research efforts have increasingly focused on employing biomass as a raw material for producing biofuels used for thermochemical conversion processes, because replacing fossil fuels with biomass would generate significant environmental benefits [4,6]. Although the thermochemical transformation of biomass is the most widely-studied strategy for converting biofuels to energy, the use of biomass on a large scale may lead to controversial competition for arable land, water, and consequently, food [6,7]. Therefore, only waste materials and agricultural by-products and residues should be used for the production of biofuels.

One of the primary by-products of agricultural production is corn stover, which consists of post-harvest corn residues [8,9]. Corn stover is an valuable and underappreciated biomass because it is abundant and widely available, both locally [7,9,10] and globally [8,11,12,13]. These harvest corn straw residues contain four key fractions, i.e., husks, leaves, cobs, and stalks, which are structurally and morphologically distinct. According to Shinners et al. [14], 1 kg of maize grain (dry weight) includes 15% of cob cores, 22% of leaves, 14% of cover leaves, and 50% of stalks. Accordingly, the higher the grain yield, the higher the mass of crop residues, and these aspects are related to the earliness of maize varieties. In the last decade the production of corn grain was highest at 6.7 mln tons in Poland. Therefore, the mass potential of corn cobs in Poland is 1.0 mln tons. The corn cobs cores may be a significant source of energy in Poland.

A digital classification of the earliness of maize varieties, known as FAO numbers, was adopted in the 1950s by the Food and Agriculture Organization of the United Nations (FAO). According to this system, the varieties are divided into nine classes of earliness marked with three figures, i.e., 100–190, 200–290, …, 900–990. The first digit indicates the basic class of earliness, the second digit represents the group of earliness as part of the basic class, and the last digit specifies the caryopsis color (zero and even numbers denote yellow caryopsis, and odd numbers denote white caryopsis).

In Poland, an additional division is used to describe the earliness of varieties: up to FAO 190—very early; FAO 200–220—early; FAO 230–240—medium-early; FAO 250–290—medium-late; and FAO 300 and above—late.

Early varieties yield less than medium-early and mid-late varieties, but they are characterized by the highest proportion of cobs and guarantee harvesting of grain 10–20 days earlier, with a moisture content of 25–30%. Therefore, an area of lack of scientific knowledge has been recognized regarding the influence of the FAO earliness pattern of maize varieties on the chemical and energetical properties of cob cores.

Due to the high value of specific heat [11] and low value of the thermal conductivity [15], corn stover biomass is used as an insulating material. However, many studies have investigated the use of corn straw residues from grain maize as a source of energy. For example, harvest residues are used to produce methane [2,7,13,16,17,18], as biomass for combustion [19,20,21,22,23], and as raw materials for bioethanol production [23,24,25,26,27]. The energetic and chemical properties of corn straw residue fractions have also been reported in the literature [22,23,28,29,30,31]. Due to their high calorific value the corn cob cores are often used as fuel, and the by-products of the combustion process are also used. The products of the corn cob cores combustion process, i.e., ash, can be used as partial substitutes in cement production. Șerbănoiu et al. [32] demonstrated that the corn cob ash content improved properties of concrete, such as resistance to chemical agents and resistance to repeated freeze–thaw cycles. The addition of corn cob cores ash reduces the compressive strength of the concrete, but it is still much higher than the requirements for the masonry structures [33,34]. However, none of these reports correlate the chemical and energetic properties of corn straw residues to the FAO’s earliness scale, despite the fact that the maize harvest time influences the crop residues properties [26,35]. For example, maize varieties with higher FAO numbers mature later, i.e., they stay longer in the field than earlier varieties. Therefore, this study aimed to determine the effect of selected maize cultivars with distinct FAO earliness classifications on the chemical and energetic properties of corn cob cores. The aim of the study was determined by practical reasons because the industrial heat installations demand fuel with homogeneous parameters. The chemical properties of corn cob cores were determined based on elemental analysis, and the energetic properties were evaluated based on the heat of combustion and calorific value. The content of ash and volatile compounds in the corn cobs was also determined.

## 2. Materials and Methods

### 2.1. Research Material

The research materials consisted of cob cores from selected corn cultivars, differing in terms of their FAO earliness. The cob cores were collected on 22 October 2019 from maize plantations located on a farm in Kiedrowo, Poland, near Wągrowiec [52.85196, 17.3860]. Corn cob cores were harvested by hand from the field. This allowed us to eliminate the contamination of the corn cobs with mineral from the soil. A total of *n* positions with an area of 1 m^2^ each were randomly marked with a frame in the field. Laboratory tests were performed on the cob cores of early (Ambrosini FAO 220), medium-early (Kampinos FAO 230 and Ricardinio FAO 240), and medium-late (Iconico FAO 250, Kidemos FAO 260, and Koletis FAO 280) varieties.

### 2.2. Determination of Moisture, Ash, and Volatile Matter Contents

The moisture content of the corn cob cores after harvesting was determined using the oven-dry method at 103 ± 2 °C. The mass measurement was performed with an accuracy of 0.1 g (OHAUS, Parsippany, NJ, USA). The cob core samples had an average mass of 200 g.

The ash content in the corn cob cores was tested in accordance with the ISO 18122 standard, “Solid biofuels—Determination of ash content…” The samples in the crucibles were heated in an air atmosphere to a temperature of 550 °C and maintained at that temperature there until the mass stabilized.

The percentage of volatile matter was determined in accordance with the ISO 18123 standard, “Solid biofuels—Determination of the content of volatile matter”.

The content of ash and volatile compounds were determined in triplicate for each option of experiment. The mass measurements were performed with an accuracy of 0.0001 g.

### 2.3. Elemental Analysis

The carbon and nitrogen content were determined using a Flash 2000 elemental analyzer (Thermo Fisher Scientific, Waltham, MA, USA) in CHNS/O configuration according to the EN 15104 standard. The instrument was calibrated with standard BBOT (2,5-bis-(5-tertbutyl-benzoxazol-2-yl)thiophene) (Thermo Fisher Scientific, Waltham, MA, USA), and the Birch leaf certified reference material (Elemental Microanalysis Ltd., Okehampton, UK) to determine the C/N ratio. Due to the negligible content of sulfur and phosphorus elements, these were not determined.

### 2.4. Combustion Heat and Calorific Value

The combustion heat tests were carried out in an automatic calorimeter (LECO AC600) calibrated with benzoic acid (LECO, New York, NY, USA) in accordance with the PN-EN ISO 18125: 2017-07 standard. To determine the heat of combustion, samples of corn cob cores from varieties with different FAO standards were used, and the moisture content was determined separately for immediately after harvesting and seasoned samples (average moisture content of 6%).

The calorific value of the corn cob cores of the studied cultivars was calculated using Equation (1),

LHV = HHV − P (W_a_ + 8.94 (H_a_)), (1)

where LHV (lower heating value) is the calorific value in the analytical state (J/g), HHV is higher heating value (J/g), P is the heat of water evaporation at 25 °C 1% content = 24.42 (J/g), Wa is the moisture content of the analytical sample (%), and Ha is the hydrogen content of the analytical sample (%).

### 2.5. Statistical Analysis

STATISTICA 13.3 software (TIBCO Software Inc., Palo Alto, CA, USA) software was used for statistical analysis of the results. The calculations included an analysis of variance (ANOVA) for the one-factor system, followed by Tukey’s HSD test (honest significant difference test) for each variable at α = 0.05.

Pearson correlations (r) among the variables were also calculated. The strength of the correlations were described using the ranges suggested by Evans [36].

## 3. Results and Discussion

### 3.1. Moisture Content of Cob Cores

The heat of combustion indicates the energy value of biomass [37], and the value of the heat of combustion of biomass depends on its moisture, chemical composition, and ash contents [38]. Therefore, determining the moisture content is a standard procedure when assessing the energy value of biomass.

The moisture content of maize grain ranged from 26.25 to 34.15% depending on the variety earliness (Table 1). The moisture content of the latest Koletis variety with FAO 280 was the highest (34.15%), and the grain moisture contents of the other varieties were similar and ranged from 26.25 to 27.15%.

The moisture of the cob cores immediately after harvest ranged from 39.12% to 57.00%. The cores of the Koletis cobs (FAO 280) had the highest moisture, and the Kidemos cobs (FAO 260) had the lowest (Table 1).

The correlations were calculated to determine the influence of the cultivar’s earliness on the moisture content of the cob cores and corn grain. The results of the calculations indicate that the FAO earliness is moderately correlated with maize grain moisture (r = 0.63) and poorly correlated with the cob core moisture (r = 0.40) (Table 2).

**Figure 1:**
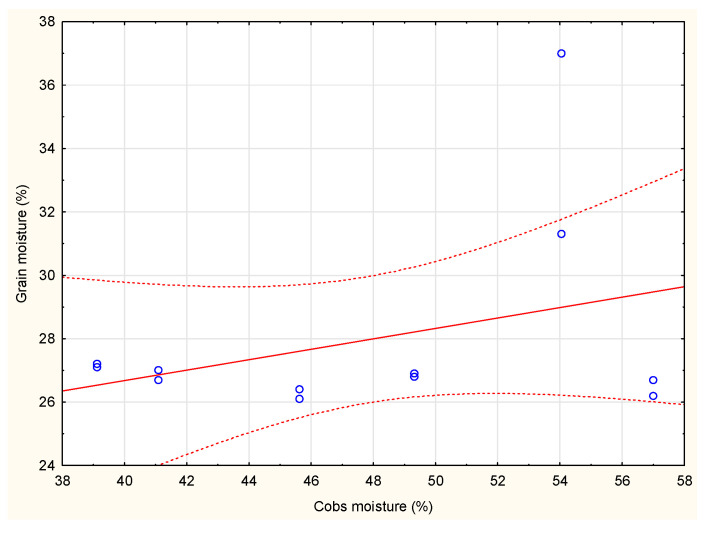
Correlation between corn cobs moisture and corn grain moisture (r = 0.34).

The correlation between grain moisture and cob cores’ moisture was very weak (r = 0.34) (Figure 1). The correlation between grain and corn cob moisture shows a non-obvious correlation. Moisture content of FAO 260 maize was equal to 27.2% and 39.1 for grain and cobs, respectively. The obtained values of moisture content for FAO 260 were similar to maize, with the lowest value being FAO 220. For FAO 250 maize we determined the lowest moisture value for grain and the highest for cobs.

### 3.2. Ash and Volatile Matter Content

Ash content is a crucial parameter directly affecting the heat of biomass combustion [37,39]. High ash content in plant matter or biomass makes it less desirable as a fuel [39].

The ANOVA test results indicated that the ash content in the cob cores differed depending on the maize variety’s FAO earliness factor. The highest ash content was found in the cores of maize cobs with FAO 280 (2.58%), whereas the lowest ash content was found in the maize cob cores of FAO 250 (1.04%). The cultivars with FAO number 280, 260, and 240 formed a homogeneous group in terms of their ash content, which was significantly higher than that of the other analyzed varieties (Table 3).

For comparison, Maj et al. [22] found that the ash content in corn cob cores obtained from the seed plant was 5.64%; however, the authors did not provide the variety or FAO earliness of the maize for the cob cores on which they conducted the study. Moreover, the method of harvesting the corn cob cores was not provided.

Lizotte et al. [40] determined the ash content in the cob cores of two varieties of Elite 46T07 and Elite 30A27 maize grown in two temperature zones in Canada. According to the FAO classification, these varieties were considered as early (FAO 180–210). According to these authors, the ash content in corn cob cores grown in the cooler zone averaged 2.07%, and in the warmer zone it averaged 2.26%. These values are similar to the results obtained in the present research.

Wojcieszak et al. [35] conducted a study of the ash content in the corn cob cores with FAO 220, depending on the harvest maturity. The authors reported that the ash content in the cob cores was 2% in the optimal harvest time and the same amount in the delayed date.

Louis and Venkatachalam [41] reported that the ash content of Indian corn cob cores was 3.1%, but they did not provide the variety and earliness FAO number. Ahmad and Subawi [42] also reported that the ash content in corn cob cores were between 2.8% and 1.4%, without assigning values to the varieties. Takada et al. [43] separated the core of the corn cobs into three fractions, i.e., chaff, woody ring, and pith, which accounted for 21.1%, 77.5%, and 1.4% of the core dry weight, respectively. The authors stated that the ash contents in individual fractions were 3.1%, 1.4%, and 2.4%, respectively.

Yin [38] reported a much higher ash content in maize crop residues (6.73%) but did not provide the FAO number or source of the research material. A similar ash content in maize residue (3.07–7.40%) was determined in a study conducted by Xiong et al. [44].

Volatile matter is a characteristic feature of solid fuels, which is standardized when assessing energy biomass [45,46,47]; biomass is characterized by a high content of volatile matter [46], which consists of gaseous products and vapors formed during the thermal decomposition of solid fuel under anaerobic conditions. The quantity of volatile components essential for assessing solid fuel’s energy suitability decreases as its carbonization degree increases. Fuels containing numerous volatile components generate a long flame during combustion and require an additional air supply for complete smoke-free combustion.

The mean content of volatile matter in the analyzed corn cob cores was 94.5–91.3%, depending on the FAO number of a given variety.

For comparison, Maj et al. [21] indicated that the content of volatile compounds in the corn cobs cores from seed production was 69.24%, which was lower than the values obtained in the analyses performed herein. Wang et al. [42] measured the volatile matter content of maize stalks and found that it was 67.64%. In contrast, Lu et al. [43] conducted studies of the volatile matter content in 66 types of biomass and reported that their minimum content was 70.39% and the maximum content was 83.92%. This discussion shows that the volatile components reported in the literature are generally lower than the values obtained in the tests conducted in the present study.

### 3.3. Elemental Analysis

Elemental analysis involves assessing the essential elements comprising the organic matter in solid fuels, i.e., C, H, N. The organic matter mainly consisted of these chemical elements, while other elements were present in negligible amounts (phosphorus, chlorine, sulfur). Elemental analysis is commonly used to evaluate energetic biomass [48,49].

Elemental analysis of the tested cob cores from maize cultivars differing in FAO earliness involved determining the carbon content (as a%). The carbon content in the corn cob cores ranged from 38.50 to 44.08%, depending on the FAO earliness number. The highest carbon content was found in maize cob cores with FAO 220, and the lowest was found in maize cob cores with FAO 260. The carbon content in these cultivars’ cob cores differed significantly from that in cob cores of the other varieties analyzed in this study. The cores of maize cobs with FAO 240 contained 42.59% C, which differed significantly from the other cultivars (α = 0.05) (Table 4).

Maj et al. [22] indicated that the carbon content in corn cob cores was 48.51%, which was higher than the results obtained herein. The authors also reported that the percentage of carbon in the cob’s leaves was 31.06%, which was less than in the cores. However, the mixture of cob cores and cover leaves in a proportion of 4:1 contained 47.35% C. Aseffe et al. [50] determined that the average carbon content of Ecuadorian corn cob cores was 47.82%, but they did not state the earliness pattern for that variety. Klass et al. [51] reported that the carbon content in cob cores of corn harvested in Ontario, Canada, was 36.4%, which was similar to the cob cores’ carbon content of the FAO 260 variety, as determined in this study. Wang et al. [46] found that the carbon content of maize stalks was 40.90%; however, the authors did not provide information about the variety.

The results of elemental analysis also showed statistically significant (for α = 0.05) differences in terms of the hydrogen content in different earliness varieties. The highest hydrogen content was found in maize cob cores with FAO 220 (6.00%), while the lowest was found in the variety with FAO 260 (5.09%). The cores of maize cobs with FAO 220,230, 240, 250, and 280 formed a homogeneous group in terms of hydrogen content (Table 4).

The nitrogen content in the cores of corn cobs differed significantly (α = 0.05) depending on maize’s FAO earliness number. The highest nitrogen content was found in maize cob cores with the latest FAO 280 (0.80%), while the smallest nitrogen content was found in cores of maize cobs with the earliest FAO 220. The cores of the cobs from FAO 240 contained 0.78% nitrogen, which was similar to that in the cob cores from FAO 280 (Table 4).

### 3.4. Combustion Heat and Calorific Value

The value of biomass during the combustion process can be assessed using two indicators. The first indicator is the heat of combustion (HHV), which is defined as the amount of heat released during complete combustion when burning a unit of mass or volume of fuel (assuming that the exhaust gas reaches the fuel’s initial temperature and that the state of their aggregate remains stable throughout the thermodynamic measurements). The second indicator is the calorific value (LHV), which is defined as the amount of heat released during complete combustion when burning a unit of mass or volume of fuel (assuming that the water vapor contained in the exhaust gas does not condense, although the exhaust gas reaches the initial fuel temperature) [52].

The research scope of the present study included determining the heat of combustion in the isoperibol calorimeter of the cob cores in the fresh state, immediately after harvesting, and after drying to an average moisture content of 6%. The calorific value was then determined based on the heat of combustion measurements.

The heat of combustion of the cob cores in the fresh state ranged from 7.62 to 10.79 MJ/kg, depending on the variety (Table 5). The highest heat of combustion was found for the cob cores with FAO 260. However, Pearson’s analysis indicated a very weak correlation between the heat of combustion value and the variety (r = −0.36). The analysis showed a very strong correlation (r = −1.0) between the heat of combustion value and the moisture content (Table 7).

The calorific value of corn cob cores immediately after harvesting ranged from 5.02 to 8.75 MJ/kg. ANOVA analysis indicated that the differences between the calorific values of the cob cores were statistically significant (Table 5). However, the calculated value of Pearson’s coefficient indicated that the correlation between the calorific value and the heat of combustion was very weak (r = −0.32), but the correlation between the calorific value and moisture content was very strong (r = −1.0) (Table 6).

The value of the heat of combustion of seasoned corn cob cores (average moisture content ca. 6%) ranged from 17.58 to 17.79 MJ/kg. ANOVA analysis showed that depending on the cultivar’s earliness pattern, the cob cores formed two homogeneous groups in terms of combustion heat (Table 7).

The calorific value of the seasoned corn cob cores ranged from 16.19 to 16.53 MJ/kg, depending on the variety. The calorific value of the corn cob cores of the maize variety with FAO 220 was 16.19 MJ/kg, and the calorific value of the cores from the FAO 280 variety was 16.21 MJ/kg. The difference between these values was not statistically significant (Table 6).

For comparison, Maj et al. [22] found that the combustion heat of cob cores with a moisture content of 7.83% was 17.05 MJ/kg, while the calorific value was 14.94 MJ/kg. Miranda et al. [53] determined that the calorific value of pellets made from corn cob cores with 12% moisture was 14.5 MJ/kg. Martillo et al. [50] found that the heat of combustion of the cores of corn cobs was 19.34 MJ/kg, and the calorific value was 17.79 MJ/kg. These values were higher than the results obtained in this study, but the authors reported that they were calculated on the basis of elemental composition.

Maj et al. [22] found that the heat of combustion of corn cobs with a moisture content of 9.49% was 10.96 MJ/kg, and the calorific value was 9.69 MJ. Channiwala and Parikh [54] reported that the heat of combustion of wheat straw was 17.99 MJ/kg, and the heat of combustion of rice hulls was 14.69 MJ/kg. Moreover, Mory et al. [55] found that the heat of maize crop residues combusted in the dry state was 17.93 MJ/kg. Tortas et al. [52] reported similar value of the heat combustion equal to 17.68 MJ/kg for maize crop residues. For oven dry Miscanthus biomass, Danielewicz et al. [53] determined the mean heating value equal to 18.56 MJ/kg. Taking into account the 6% of moisture content of the analyzed corn cob cores, it can be considered a valuable fuel.

## 4. Conclusions

Based on the results of these investigations, the following main conclusions were drawn:

1. The FAO earliness of a maize variety had a significant impact on the elemental composition, ash content and calorific value in the cob cores.
2. The FAO earliness standard is an indicator for determining the calorific value of corn cob cores. Correlation between maize cultivar earliness FAO with core moisture content was found.
3. Corn cob cores moisture depends on variety earliness but is not the highest for varieties with the highest FAO. This is important information for energy producers as well as for seasoning processes in order to reduce moisture content of corn cob cores.
4. The obtained high calorific values of corn cob cores indicate their suitability as a fuel in biomass combustion processes.

## Figures and Tables

**Table 1:** Average moisture content of grain and corn cob cores at harvest.

Corn Variety	Average Grain Moisture (%)	Average Cob Cores Moisture (%)
FAO 220	26.9 ± 0.2	41.1 ± 0.1
230	26.3 ± 0.2	45.6 ± 0.4
FAO 240	26.9 ± 0.1	49.3 ± 0.2
FAO 250	26.5 ± 0.4	57.0 ± 0.3
FAO 260	27.2 ± 0.1	39.1 ± 0.1
FAO 280	34.2 ± 0.9	54.1 ± 0.3
*n*	2	2

± std ± standard deviation.

**Table 2:** Correlation between the FAO earliness and the moisture of grain and cob cores.

	Variety	Grain Moisture
Grain moisture	0.63	-
Cobs moisture	0.40	0.34

**Table 3:** Ash and volatile matter contents.

Corn Variety	Ash (%)	Volatile Parts (%)
FAO 220	1.31 ^c^ ± 0.31	93.8 ^ab^ ± 0.4
FAO 230	1.89 ^bc^ ± 0.42	91.3 ^b^ ± 1.5
FAO 240	2.12 ^abc^ ± 0.28	93.6 ^ab^ ± 0.8
FAO 250	1.04 ^d^ ± 0.46	92.1 ^ab^ ± 1.3
FAO 260	2.58 ^ab^ ± 0.14	94.1 ^a^ ± 0.3
FAO 280	2.67 ^a^ ± 0.26	94.5 ^a^ ± 1.3
*n*	4	4

Numbers presented as average values (*n*) ± standard deviation; the same superscripts (a, b, c, d, e, f) indicate that a considerable difference was not found between average values in columns according to the HSD Tukey test (ANOVA) for the investigated factors.

**Table 4:** Elemental composition of cob cores depending on the FAO earliness pattern.

Corn Variety	C	H	N
FAO 220	44.08 ^a^ ± 0.52	6.00 ^a^ ± 0.29	0.56 ^d^ ± 0.02
FAO 230	43.47 ^ab^ ± 0.24	5.63 ^ab^ ± 0.17	0.64 ^c^ ± 0.02
FAO 240	42.59 ^c^ ± 0.05	5.65 ^ab^ ± 0.14	0.78 ^a^ ± 0.00
FAO 250	43.34 ^abc^ ± 0.09	5.65 ^abc^ ± 0.11	0.66 ^c^ ± 0.01
FAO 260	38.50 ^d^ ± 0.70	5.09 ^c^ ± 0.06	0.73 ^b^ ± 0.02
FAO 280	42.92 ^bc^ ± 0.11	5.64 ^b^ ± 0.12	0.80 ^a^ ± 0.03
*n*	4	4	4

Numbers are reported as average values (*n*) ± standard deviation; the same superscripts (a, b, c, d,) indicate that a considerable difference (α = 0.05) was not determined between average values in these columns according to the HSD Tukey test (ANOVA) for the investigated factors.

**Table 5:** Heat of combustion and calorific value of corn cob cores after harvest depending on the FAO number.

Corn Variety	Average HHV (MJ/kg)	Average LHV (MJ/kg)
FAO 220	10.38 ^c^ ± 0.01	8.10 ^b^ ± 0.07
FAO 230	9.60 ^bc^ ± 0.02	7.28 ^c^ ± 0.05
FAO 240	9.02 ^ab^ ± 0.04	6.60 ^d^ ± 0.06
FAO 250	7.62 ^abc^ ± 0.02	5.02 ^f^ ± 0.02
FAO 260	10.79 ^a^ ± 0.08	8.75 ^a^ ± 0.10
FAO 280	8.08 ^c^ ± 0.02	5.57 ^e^ ± 0.06
*n*	4	4

Numbers are reported as average values (*n*) ± standard deviation; the same superscripts (a, b, c, d, e, f) indicate that a considerable difference (α = 0.05) was not determined between average values in these columns according to the HSD Tukey test (ANOVA) for the investigated factors.

**Table 6:** Pearson’s correlation coefficients between corn varieties, chemical composition, and heat values.

	Corn Varieties	C	H	N	Ash	VM	Grain Moisture after Harvest	HHV
C	−0.48	-	-	-	-	-	-	-
H	−0.60	0.82	-	-	-	-	-	-
N	0.75	−0.33	−0.42	-	-	-	-	-
Ash	0.55	−0.52	−0.49	0.70	-	-	-	-
WM	0.39	−0.30	−0.03	0.40	0.49	-	-	-
Moisture after harvest	0.36	0.53	0.26	0.33	−0.23	−0.14	-	-
HHV	−0.36	−0.54	−0.27	−0.32	0.23	0.14	−1.00	-
LHV	−0.32	−0.57	−0.32	−0.30	0.25	0.14	−1.00	1.00

**Table 7:** Combustion heat and calorific value of seasoned corn cob cores.

Corn Variety	Average HHV (MJ/kg)	Average LHV (MJ/kg)
FAO 220	17.63 ^b^ ± 0.02	16.19 ^c^ ± 0.07
FAO 230	17.65 ^ab^ ± 0.04	16.28 ^bc^ ± 0.07
FAO 240	17.79 ^a^ ± 0.08	16.45 ^ab^ ± 0.09
FAO 250	17.72 ^ab^ ± 0.04	16.36 ^abc^ ± 0.05
FAO 260	17.72 ^ab^ ± 0.12	16.53 ^a^ ± 0.15
FAO 280	17.58 ^b^ ± 0.04	16.21 ^c^ ± 0.08
*n*	4	4

Numbers are reported as average values (*n*) ± standard deviation; the same superscripts (a, b, c,) indicate that a considerable difference (α = 0.05) was not determined between average values in these columns according to the HSD Tukey test (ANOVA) for the investigated factors.
